# Antibiotic Resistance Profiles of *Haemophilus influenzae* Isolates from Children in 2016: A Multicenter Study in China

**DOI:** 10.1155/2019/6456321

**Published:** 2019-08-14

**Authors:** Hong-Jiao Wang, Chuan-Qing Wang, Chun-Zhen Hua, Hui Yu, Ting Zhang, Hong Zhang, Shi-Fu Wang, Ai-Wei Lin, Qing Cao, Wei-Chun Huang, Hui-Ling Deng, Shan-Cheng Cao, Xue-jun Chen

**Affiliations:** ^1^Division of Infectious Diseases, The Children's Hospital, Zhejiang University School of Medicine, Hangzhou 310003, China; ^2^Department of Clinical Laboratory, Children's Hospital of Fudan University, Shanghai 201102, China; ^3^Division of Infectious Diseases, Children's Hospital of Fudan University, Shanghai 201102, China; ^4^Division of Infectious Diseases, Children's Hospital of Shanghai Jiaotong University, Shanghai 200240, China; ^5^Department of Clinical Laboratory, Children's Hospital of Shanghai Jiaotong University, Shanghai 200240, China; ^6^Department of Clinical Laboratory, Qilu Children's Hospital of Shandong University, Jinan 250022, China; ^7^Division of Infectious Diseases, Qilu Children's Hospital of Shandong University, Jinan 250022, China; ^8^Division of Infectious Diseases, Shanghai Children's Medical Center, Shanghai 200127, China; ^9^Department of Clinical Laboratory, Shanghai Children's Medical Center, Shanghai 200127, China; ^10^Department of Clinical Laboratory, Xi'an Children's Hospital, Xi'an 710043, China; ^11^Division of Infectious Diseases, Xi'an Children's Hospital, Xi'an 710043, China; ^12^Department of Clinical Laboratory, The Children's Hospital, Zhejiang University School of Medicine, Hangzhou 310003, China

## Abstract

**Background and Objective:**

*Haemophilus influenzae* (HI) is a common cause of community-acquired pneumonia in children. In many countries, HI strains are increasingly resistant to ampicillin and other commonly prescribed antibiotics, posing a challenge for effective clinical treatment. This study was undertaken to determine the antibiotic resistance profiles of HI isolates from Chinese children and to provide guidelines for clinical treatment.

**Methods:**

Our Infectious Disease Surveillance of Pediatrics (ISPED) collaboration group includes six children's hospitals in different regions of China. The same protocols and guidelines were used by all collaborators for the culture and identification of HI. The Kirby–Bauer method was used to test antibiotic susceptibility, and a cefinase disc was used to detect *β*-lactamase activity.

**Results:**

We isolated 2073 HI strains in 2016: 83.9% from the respiratory tract, 11.1% from vaginal secretions, and 0.5% from blood. Patients with respiratory isolates were significantly younger than nonrespiratory patients (*P* < 0.001). Of all 2073 strains, 50.3% were positive for *β*-lactamase and 58.1% were resistant to ampicillin; 9.3% were *β*-lactamase-negative and ampicillin-resistant. The resistance rates of the HI isolates to trimethoprim-sulfamethoxazole, azithromycin, cefuroxime, ampicillin-sulbactam, cefotaxime, and meropenem were 71.1%, 32.0%, 31.2%, 17.6%, 5.9%, and 0.2%, respectively.

**Conclusions:**

More than half of the HI strains isolated from Chinese children were resistant to ampicillin, primarily due to the production of *β*-lactamase. Cefotaxime and other third-generation cephalosporins could be the first choice for the treatment of ampicillin-resistant HI infections.

## 1. Introduction

*Haemophilus influenzae* (HI) is one of the most common pathogens that cause community-acquired pneumonia and otitis media in children [1–4]. HI also causes pediatric invasive diseases, such as meningitis and sepsis [5–7]. These HI-related diseases seriously threaten child health. At the beginning of the 1970s, ampicillin became the drug of choice for the treatment of HI infections. However, as antibiotic usage has become increasingly widespread globally, the resistance of HI strains to ampicillin has also gradually increased [8–11]. This resistance rate varies greatly among different countries and regions [8–11]. In 882 Canadian HI strains, the ampicillin resistance rate was 13.5% between 2007 and 2014, but increased to 19% in the following four years [8]. Similarly, the ampicillin resistance rate of Japanese HI strains increased from 28.8% (2000–2001) to 63.5% (in 2012) [9]. Between 2012 and 2014, the ampicillin resistance rates of HI strains isolated in India, Singapore, Thailand, and South Korea were 7.4%, 21.7%, 39.5%, and 69.4%, respectively [10]. The ampicillin resistance rate of HI strains in China was 19.9% between 2009 and 2011 (based on 307 HI strains) and 32.4% between 2013 and 2014 (based on 185 HI strains) [11]. The increase of ampicillin resistance rate of HI posed a challenge for effective therapy. This study was undertaken to determine the antibiotic resistance profiles of HI isolates from Chinese children in 2016 and to provide guidelines for clinical treatment. Given the vast size of China, a large-scale multicenter study was conducted to characterize HI infection and antibiotic resistance in Chinese children.

## 2. Materials and Methods

### 2.1. Strain Isolation and Identification

The ISPED collaboration group includes six children's hospitals in different regions of China: Children's Hospital, School of Medicine, Zhejiang University, Hangzhou, Zhejiang; Children's Hospital of Fudan University, Shanghai; Shanghai Children's Medical Center, Shanghai; Qilu Children's Hospital of Shandong University, Jinan, Shandong; Children's Hospital of Shanghai Jiaotong University, Shanghai; and Xi'an Children's Hospital, Xi'an, Shanxi. Clinical isolates were collected and identified between January 2016 and December 2016. Using identical protocols, different haemophilus specimens were inoculated in haemophilus selective medium (Mérieux, France) and incubated in 5% CO_2_ for 18–24 h. Clear or translucent, flat, moist, dew-drop colonies were selected, confirmed as Gram-negative, and identified using the Vitek system NH card (Mérieux, France).

### 2.2. *β*-Lactamase Detection and Susceptibility Testing

*β*-lactamase production was measured using the cefinase disc method (BioMériex, France). The Kirby–Bauer method was used to test drug susceptibility, following the 2016 American Committee for Clinical Laboratory Standards (CLSI) guidelines M100-S26 [12]. Susceptibility to the following antibiotics was tested: ampicillin (10 *μ*g), ampicillin-sulbactam (10 *μ*g/10 *μ*g), cefuroxime (30 *μ*g), cefotaxime (30 *μ*g), meropenem (10 *μ*g), chloramphenicol (30 *μ*g), trimethoprim-sulfamethoxazole (TMP-SMZ) (1.25/23.75 *μ*g), levofloxacin (5 *μ*g), and azithromycin (15 *μ*g) (Oxoid, UK). HI strain ATCC49247 was used for quality control throughout the test.

### 2.3. Statistical Methods

Comparisons of antibiotic resistant rate between groups were performed with the χ^2^ test. Age data, which were nonnormally distributed, were described as medians, and nonparametric tests were used for group-group comparisons. *P* values < 0.05 were considered statistically significant.

## 3. Results

### 3.1. Sources of Isolates

**Figure 1:**
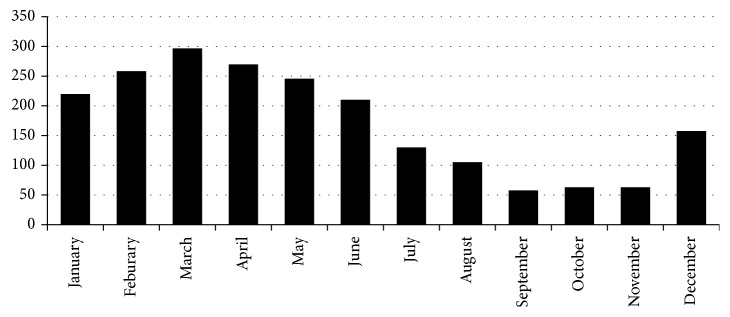
Seasonal distribution of 2073 *Haemophilus influenzae* strains isolated from six Chinese hospitals in 2016.

We isolated 2073 HI strains in 2016. Of these, 1734 (83.6%) were from respiratory tract samples: 1558 from sputum, 107 from throat swabs, 60 from alveolar lavage fluid, and 9 from lung puncture fluid. The remaining 339 HI strains (16.4%) were from nonrespiratory tract samples: 231 from vaginal swabs, 48 from eye secretions, 37 from middle ear effusions, 10 from blood, 2 from cerebrospinal fluid, and 11 from other sources. We collected 1171 of the HI strains (56.5%) from males. The patients ranged in age from 10 d to 15 y (median: 2.19 y). The median age of the respiratory patients (0.92 y) was significantly lower than that of nonrespiratory patients (5.0 y; *Z* = 18.32; *P* < 0.001). HI cases were unevenly distributed throughout the year, with 1498 HI strains (72.3%) isolated between January and June (Figure 1); the number of HI strains peaked in March (297 HI strains; 14.3%; Figure 1).

### 3.2. *β*-Lactamase Detection and Antibiotic Susceptibility

Of the 2073 HI strains isolated, 1042 (50.3%) were positive for *β*-lactamase. The ampicillin resistance rate across all strains was 58.1%: 193 strains (9.3%) were *β*-lactamase-negative and ampicillin-resistant (BLNAR), while 160 strains (7.7%) were *β*-lactamase-positive and ampicillin-sulbactam-resistant (Table 1). *β*-lactamase-positive HI strains had significantly higher resistance rates to chloramphenicol, trimethoprim-sulfamethoxazole, and azithromycin than *β*-lactamase-negative strains did (*P* < 0.01; Table 2). The resistance rates of respiratory tract-derived HI strains to ampicillin, ampicillin-sulbactam sodium, cefuroxime, TMP-SMZ, and azithromycin were higher than those of nonrespiratory strains (*P* < 0.01; Table 3).

### 3.3. Multidrug Resistance

Among 1617 HI isolates for which all antibiotics-resistance data were available, 520 (32.2%) were resistant to more than three different types of antibiotics. The most common multidrug resistance pattern was resistance to ampicillin, sulfamethoxazole, and azithromycin. This pattern was observed in 441 HI strains (84.8% of all multidrug-resistant strains). An additional 11 HI strains (2.1% of all multidrug-resistant strains) were resistant to ampicillin, chloramphenicol, sulfamethoxazole, and azithromycin.

## 4. Discussion

The majority of the HI isolates identified here were obtained from the sputa of children with lower respiratory tract infections. This supported previous suggestions that HI is one of the most common causes of community-acquired pneumonia in children [13]. An additional 11.1% of the HI strains isolated here were obtained from vaginal swabs of children with symptomatic vaginitis, suggesting that HI is also a common cause of vulvovaginal infection in prepubescent girls [13]. Infants, especially those less than 6 months old, have very low levels of HI antibodies in their blood [14] and are therefore highly susceptible to HI infection [3, 6, 13]. Infants infected with HI commonly develop pneumonia [13]. HI strains that cause vaginal inflammation may be of respiratory origin [15]. Here, patients with vaginally derived HI isolates were significantly older than those with simple respiratory infections, which is consistent with our previous study [13]. This might be because older children more easily transmit bacteria among body parts through movements of the mouth and hands. The invasive HI infection rate was low in this study: only 10 HI strains were isolated from blood and only 2 were from cerebrospinal fluid. Treatment with effective antibiotics prior to specimen collection may have contributed to the low detection of HI strains in the blood. The rates of HI infections in children identified here fluctuated obviously with season. However, seasonal distributions may vary in different regions or in different years due to changes in climatic conditions. Here, most HI isolates were collected between February and May, consistent with a previous study in our hospital in 2015 [13].

Here, the HI ampicillin resistance rate was 58.1%, higher than what have been found in one previous Chinese study (19.9–32.4%, 2009–2014) [11] and two European studies (29.1% in Poland and 11.6% in Germany) [16, 17], but close to ampicillin resistance rates in Japan [9] and South Korea [10] (63.5–69.4%). The main mechanism used by HI to resist ampicillin is the production of *β*-lactamase to break antibiotics. Another mechanism is the mutation of penicillin-binding protein 3 (PBP3), which decreases the antibiotic susceptibility of HI towards ampicillin and other *β*-lactam antibiotics. The *β*-lactam resistance phenotype mediated by the second mechanism is named *β*-lactamase-nonproducing ampicillin resistance (BLNAR). Although producing *β*-lactamase is the primary mechanism of ampicillin resistance [8, 13, 16], BLNAR strains are the most frequently isolated, and *β*-lactamase-producing HI are rare in Japan [9]. Of the 2073 HI strains isolated here, 50.3% were positive for *β*-lactamase, while BLNAR strains were 9.3%. This finding was consistent with previous reports [11, 13]. BLNAR strains are expected to be resistant to ampicillin-sulbactam and amoxicillin-clavulanic acid [13], while *β*-lactamase-positive HI strains are usually sensitive to these *β*-lactamase-inhibiting compounds [9, 11, 13]. Recently, *β*-lactamase-producing HI isolates with PBP3 mutations were identified, and the antibiotic resistance profiles of these isolates were similar with that of BLNAR [9]. Indeed, 7.7% of all HI strains examined here were *β*-lactamase positive and nonsusceptible to ampicillin-sulbactam; in South Korea, 40.5% of all HI strains were *β*-lactamase positive and ampicillin-sulbactam nonsusceptible [10]. In such *β*-lactamase-producing strains, ampicillin-sulbactam resistance is primarily conferred by *fts* gene mutations, which alter the amino acid sequence of the encoded PBP3 protein [9]. It is clear that antibiotic resistance patterns and mechanisms vary tremendously among different regions.

HI strains are highly susceptible to third-generation cephalosporins. Kiedrowska et al. assessed antibiotics susceptibility in vitro against 117 Polish HI isolates in 2012 and found that all of the isolates, including *β*-lactamase-positive HI and BLNAR isolates, were sensitive to ceftriaxone [17]; BLNAR HI with PBP 3 mutations or HI with *β*-lactamase production may be associated with elevated MIC values of third-generation cephalosporins, but the level did not transgress the resistance breakpoint in most isolates [16–18]. In the present study, the susceptibility rate of HI to cefotaxime/ceftriaxone was as high as 94.1%, which was in accordance with the findings in previous studies [13, 17]. These results suggested that cefotaxime or ceftriaxone might be effective for the treatment of HI infections and should become the main empirical antibiotic for therapy [19]. The meropenem susceptibility rate was even higher, up to 99.8% in the study. However, meropenem and other carbapenems should not be considered for the treatment except for life-threatening invasive HI infections or for those with clinical failure after ceftriaxone or cefotaxime treatment [20]. *β*-lactamase-positive HI strains showed resistance towards chloramphenicol, azithromycin, and sulfamethoxazole at a significantly higher rate than did *β*-lactamase-negative HI strains. The rate of multidrug resistance was also significantly higher in *β*-lactamase-positive HI strains than in *β*-lactamase-negative strains. Indeed, multidrug resistance might be conveyed by transferrable plasmid conjugative resistance elements [21–23].

HI also causes urinary and reproductive tract infections [24]. However, even within the same population in the same region, HI resistance profiles vary greatly depending on infection site [24]. In Japan, the proportion of BLNAR strains in the HI of respiratory tract was significantly higher than that of the urinary tract [24]. Notably, the majority of the ampicillin-resistant HI strains in Japan were BLNAR [9, 25]. Our research group has previously compared the antibiotic resistance of vaginal isolates to respiratory isolates in children and found that respiratory isolates were resistant to ampicillin, cefuroxime, clarithromycin, and trimethoprim-sulfamethoxazole at significantly higher rates than were vaginal isolates [13]; the results of this previous study were consistent with the present multicenter study. Collectively, our results indicated that the best antibiotic treatment for HI might vary depending on the site of infection.

A limitation of this study was the lack of serotyping data for the isolates. With the widespread use of the Hib vaccine, the number of b-type HI infections has gradually decreased, while infections caused by nontypeable unencapsulated HI strains have gradually increased [26, 27]. The *β*-lactamase-positive rate is higher in unencapsulated strains than in encapsulated strains [8]. In future multicenter surveillance studies of antibiotic resistance in HI, we will also explore the associations between the drug resistance and HI serotype to provide better information for the prevention and treatment of HI infections.

## Figures and Tables

**Table 1:** Antibiotic resistance of 2073 *Haemophilus influenzae* strains isolated from patients in six children's hospitals in China in 2016.

Antibiotic	*n*	*S* (%)	*I* (%)	*R* (%)
Ampicillin	2073	37.3	4.6	58.1
Ampicillin-sulbactam	2073	82.4	0.0	17.6
Cefuroxime	2073	63.6	5.2	31.2
Cefotaxime	2069	94.1	0.0	5.9
Meropenem	2073	99.8	0.0	0.2
Chloramphenicol	2009	92.4	3.1	4.5
Sulfamethoxazole-trimethoprim	2073	27.3	1.6	71.1
Levofloxacin	2073	99.1	0.0	0.9
Azithromycin	1617	68.0	0.0	32.0

*Note*. *S*, susceptible; *I*, intermediate; *R*, resistant.

**Table 2:** Antibiotic resistance rates of *β*-lactamase-positive and *β*-lactamase-negative isolates of *Haemophilus influenzae*.

Antibiotic	*β*-Lactamase (+)				*β*-Lactamase (−)			
	*n*	*R* (%)	*I* (%)	*S* (%)	*n*	*R* (%)	*I* (%)	*S* (%)
Ampicillin^*∗*^	1042	97.0	3.0	0.0	1031	18.7	6.3	75.0
Ampicillin-sulbactam^*∗*^	1042	15.4	0.0	84.6	1031	19.9	0.0	80.1
Cefuroxime^*∗*^	1042	41.6	0.0	51.4	1031	20.8	3.3	75.9
Cefotaxime	1039	6.4	0.0	93.6	1030	5.5	0.0	94.5
Meropenem	1042	0.2	0.0	99.8	1031	0.0	0.0	100
Chloramphenicol^*∗*^	1019	6.3	2.6	91.2	990	2.6	3.7	93.7
SXT^*∗*^	1042	79.8	1.2	18.9	1031	62.2	2.0	35.8
Levofloxacin	1042	0.9	0.0	99.1	1031	0.9	0.0	99.1
Azithromycin^*∗*^	815	55.2	0.0	44.8	802	8.5	0.0	91.5

*Note*. SXT, sulfamethoxazole-trimethoprim. ^*∗*^*P* < 0.01. *S*, susceptible; *I*, intermediate; *R*, resistant.

**Table 3:** Antibiotic resistance rates of respiratory and nonrespiratory isolates of *Haemophilus influenzae*.

Antibiotics	Respiratory				Nonrespiratory			
	*n*	*R* (%)	*I* (%)	*S* (%)	*n*	*R* (%)	*I* (%)	*S* (%)
Ampicillin^*∗*^	1734	61.4	4.8	33.8	336	41.4	3.6	55.0
Ampicillin-sulbactam^*∗*^	1734	18.3	0.0	81.7	336	14.0	0.0	86.0
Cefuroxime^*∗*^	1734	33.7	5.6	60.7	336	18.5	3.0	78.5
Cefotaxime	1730	6.4	0.0	93.6	336	3.9	0.0	96.1
Meropenem	1734	0.2	0.0	99.8	336	0.0	0.0	100
Chloramphenicol	1676	4.7	2.4	92.9	330	3.6	6.4	90.0
SXT^*∗*^	1725	75.1	1.6	23.3	335	51.9	1.8	46.3
Levofloxacin	1731	1.0	0.0	99.0	336	0.0	0.0	100
Azithromycin^*∗*^	1379	35.3	0.0	64.7	235	13.2	0.0	86.8

*Note*. SXT, sulfamethoxazole-trimethoprim. ^*∗*^*P* < 0.01. *S*, susceptible; *I*, intermediate; *R*, resistant.

## Data Availability

The data used to support the findings of this study are available from the corresponding author upon request.

## References

[1] Butler D. F., Myers A. L. (2018). Changing epidemiology of *Haemophilus influenzae* in children. *Infectious Disease Clinics of North America*.

[2] Agrawal A., Murphy T. F. (2011). *Haemophilus influenzae* infections in the *H. influenzae* type b conjugate vaccine era. *Journal of Clinical Microbiology*.

[3] Kaur R., Morris M., Pichichero M. E. (2017). Epidemiology of acute otitis media in the postpneumococcal conjugate vaccine era. *Pediatrics*.

[4] Ubukata K., Morozumi M., Sakuma M. (2018). Etiology of acute otitis media and characterization of pneumococcal isolates after introduction of 13-valent pneumococcal conjugate vaccine in Japanese children. *The Pediatric Infectious Disease Journal*.

[5] Soeters H. M., Blain A., Pondo T. (2018). Current epidemiology and trends in invasive *Haemophilus influenzae* disease—United States, 2009–2015. *Clinical Infectious Diseases*.

[6] Collins S., Vickers A., Ladhani S. (2016). Clinical and molecular epidemiology of childhood invasive nontypeable *Haemophilus influenzae* disease in England and Wales. *The Pediatric Infectious Disease Journal*.

[7] Whittaker R., Economopoulou A., Dias J. G. (2017). Epidemiology of invasive *Haemophilus influenzae* disease, Europe, 2007–2014. *Emerging Infectious Diseases*.

[8] Tsang R. S. W., Shuel M., Whyte K. (2017). Antibiotic susceptibility and molecular analysis of invasive *Haemophilus influenzae* in Canada, 2007 to 2014. *Journal of Antimicrobial Chemotherapy*.

[9] Shiro H., Sato Y., Toyonaga Y., Hanaki H., Sunakawa K. (2015). Nationwide survey of the development of drug resistance in the pediatric field in 2000-2001, 2004, 2007, 2010, and 2012: evaluation of the changes in drug sensitivity of *Haemophilus influenzae* and patients’ background factors. *Journal of Infection and Chemotherapy*.

[10] Torumkuney D., Chaiwarith R., Reechaipichitkul W. (2016). Results from the survey of antibiotic resistance (SOAR) 2012–14 in Thailand, India, South Korea and Singapore. *Journal of Antimicrobial Chemotherapy*.

[11] Hu F., Zhu D., Wang F., Morrissey I., Wang J., Torumkuney D. (2016). Results from the survey of antibiotic resistance (SOAR) 2009–11 and 2013-14 in China. *Journal of Antimicrobial Chemotherapy*.

[12] Clinical and Laboratory Standards Institute (2016). *Performance Standards Antimicrobial Susceptibility Testing; M100s*.

[13] Li J.-P., Hua C.-Z., Sun L.-Y., Wang H.-J., Chen Z.-M., Shang S.-Q. (2017). Epidemiological features and antibiotic resistance patterns of *Haemophilus influenzae* originating from respiratory tract and vaginal specimens in pediatric patients. *Journal of Pediatric and Adolescent Gynecology*.

[14] Hua C.-Z., Hu W.-L., Shang S.-Q., Li J.-P., Hong L.-Q., Yan J. (2016). Serum concentrations of antibodies against outer membrane protein P6, protein D, and T- and B-cell combined antigenic epitopes of nontypeable *Haemophilus influenzae* in children and adults of different ages. *Clinical and Vaccine Immunology*.

[15] Hansen M. T., Sanchez V. T., Eyster K., Hansen K. A. (2007). Streptococcus pyogenes pharyngeal colonization resulting in recurrent, prepubertal vulvovaginitis. *Journal of Pediatric and Adolescent Gynecology*.

[16] Lâm T.-T., Claus H., Elias J., Frosch M., Vogel U. (2015). Ampicillin resistance of invasive *Haemophilus influenzae* isolates in Germany 2009–2012. *International Journal of Medical Microbiology*.

[17] Kiedrowska M., Kuch A., Żabicka D. (2017). *β*-Lactam resistance among *Haemophilus influenzae* isolates in Poland. *Journal of Global Antimicrobial Resistance*.

[18] Søndergaard A., Nørskov-Lauritsen N. (2016). Contribution of PBP3 substitutions and TEM-1, TEM-15, and ROB-1 beta-lactamases to cefotaxime resistance in *Haemophilus influenzae* and *Haemophilus parainfluenzae*. *Microbial Drug Resistance*.

[19] Szabo B. G., Lenart K. S., Tirczka T., Ostorhazi E. (2018). Clinical and microbiological characteristics of adult invasive *Haemophilus influenzae* infections: results of a 14-year single-center experience from Hungary. *Infection*.

[20] Thomas E., Guillouzouic A., Juvin M.-E. (2019). Prevalence of *Haemophilus influenzae* with alteration of PBP 3 sequence over a 1-year period in a French hospital: focus on a clinical failure after ceftriaxone treatment. *Diagnostic Microbiology and Infectious Disease*.

[21] Mohd-Zain Z., Turner S. L., Cerdeño-Tárraga A. M. (2004). Transferable antibiotic resistance elements in *Haemophilus influenzae* share a common evolutionary origin with a diverse family of syntenic genomic islands. *Journal of Bacteriology*.

[22] Chen S. T., Clowes R. C. (1987). Nucleotide sequence comparisons of plasmids pHD131, pJB1, pFA3, and pFA7 and beta-lactamase expression in *Escherichia coli*, *Haemophilus influenzae*, and *Neisseria gonorrhoeae*. *Journal of Bacteriology*.

[23] Seyama S., Wajima T., Nakaminami H., Noguchi N. (2017). Amino acid substitution in the major multidrug efflux transporter protein AcrB contributes to low susceptibility to azithromycin in *Haemophilus influenzae*. *Antimicrobial Agents and Chemotherapy*.

[24] Deguchi T., Ito S., Hatazaki K. (2017). Antimicrobial susceptibility of *Haemophilus influenzae* strains isolated from the urethra of men with acute urethritis and/or epididymitis. *Journal of Infection and Chemotherapy*.

[25] Yanagihara K., Watanabe A., Aoki N. (2017). Nationwide surveillance of bacterial respiratory pathogens conducted by the surveillance committee of Japanese Society of Chemotherapy, the Japanese Association for Infectious Diseases, and the Japanese Society for Clinical Microbiology in 2012: general view of the pathogens’ antibacterial susceptibility. *Journal of Infection and Chemotherapy*.

[26] Langereis J. D., de Jonge M. I. (2015). Invasive disease caused by nontypeable *Haemophilus influenzae*. *Emerging Infectious Diseases*.

[27] Naito S., Takeuchi N., Ohkusu M. (2018). Clinical and bacteriologic analysis of nontypeable *Haemophilus influenzae* strains isolated from children with invasive diseases in Japan from 2008 to 2015. *Journal of Clinical Microbiology*.

